# Supplementary figures and images for: Rab11A regulates dense granule transport and secretion during Toxoplasma gondii invasion of host cells and parasite replication
Source: PLoS Pathog. 2020 May 28;16(5):e1008106. doi: 10.1371/journal.ppat.1008106 (PMC7255593; doi:10.1371/journal.ppat.1008106)

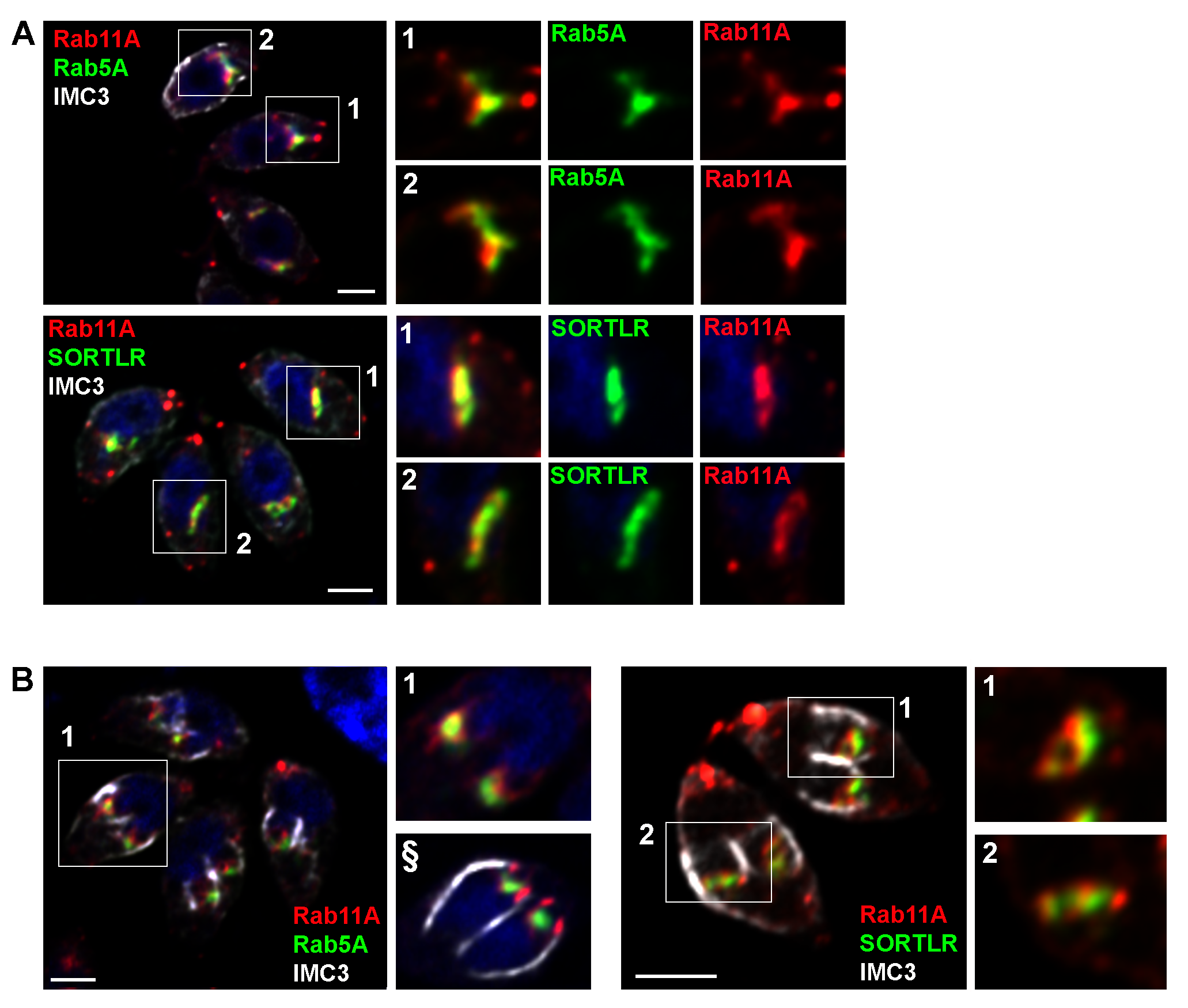

Supplement: S1 Fig — IFA showing the localization of Rab11A (red), the TGN marker TgSORTLR and the ELC marker TgRab5A (green) in fixed RHΔKU80 parasites during the G1 phase of the cell cycle (A) and cytokinesis (B). Parasite contours or daughter cells buds are revealed after detection of the protein IMC3 (white). Zooms of the areas indicated by white frames (1, 2) and corresponding to the Golgi/ELC region of a given parasite are also shown. The image B§ originating from a separate vacuole illustrates the localization of Rab11A at the tip of the forming daughter cell buds. Bars: 2 μm. (TIF) [file ppat.1008106.s001.tif]

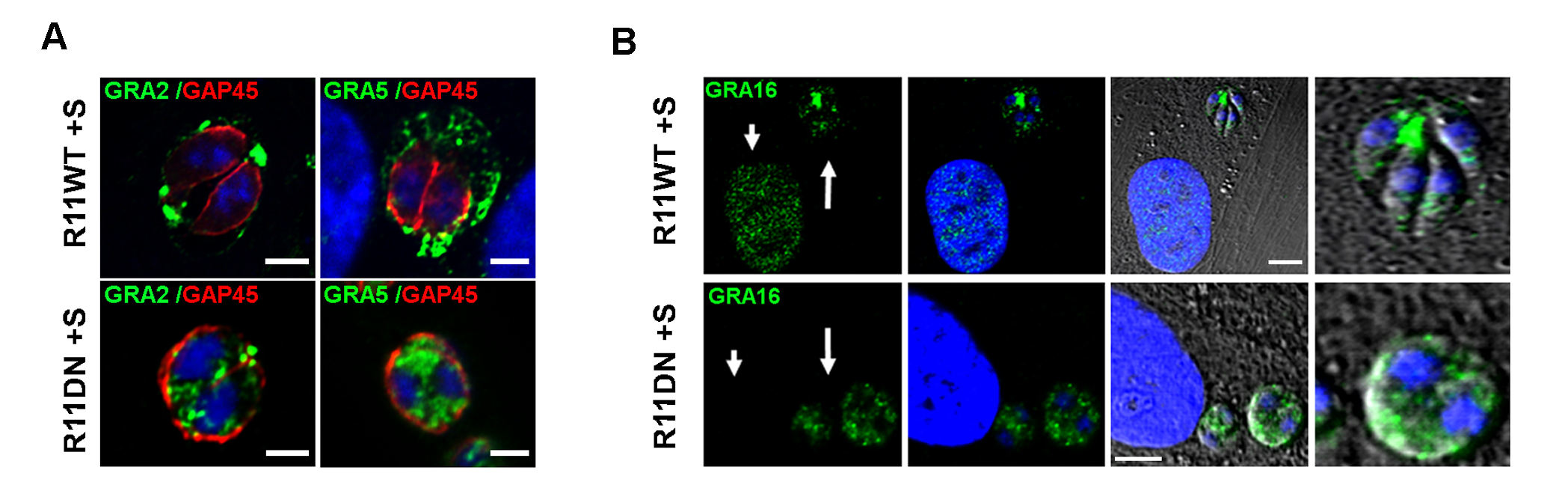

Supplement: S2 Fig — A-Immunofluorescence assay showing the dense granule proteins GRA2 and GRA5 (green) retained in intra-cytosolic vesicles in Shield-1-induced (+S) Rab11A-DN expressing parasites, while being efficiently released into the vacuolar space and at the vacuole membrane in induced Rab11A-WT expressing parasites. The parasite cortex is delineated by GAP45 (red). Bars: 2 μm. B- Fluorescence images showing the dense granule protein GRA16 (green) retained in intra-cytosolic vesicles in Shield-1-induced Rab11A-DN expressing parasites, while being secreted and translocated into the host cell nuclei (small arrows) in induced Rab11A-WT expressing parasites. Bars: 5 μm. (TIF) [file ppat.1008106.s002.tif]

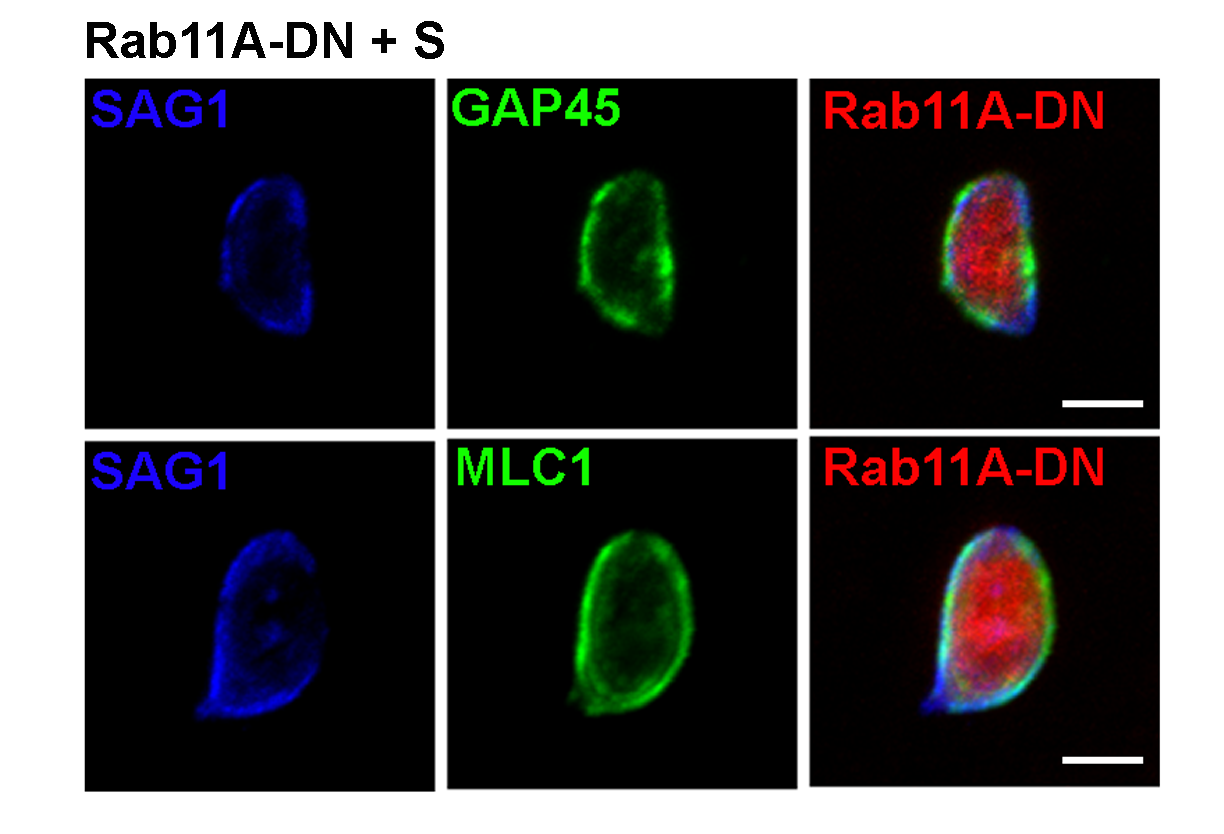

Supplement: S3 Fig — A-Immunofluorescence assay showing the cortical localization of SAG1, GAP45 and MLC1 in Shield-1-induced extracellular adherent Rab11A-DN expressing parasites. Bars: 2 μm. (TIF) [file ppat.1008106.s003.tif]

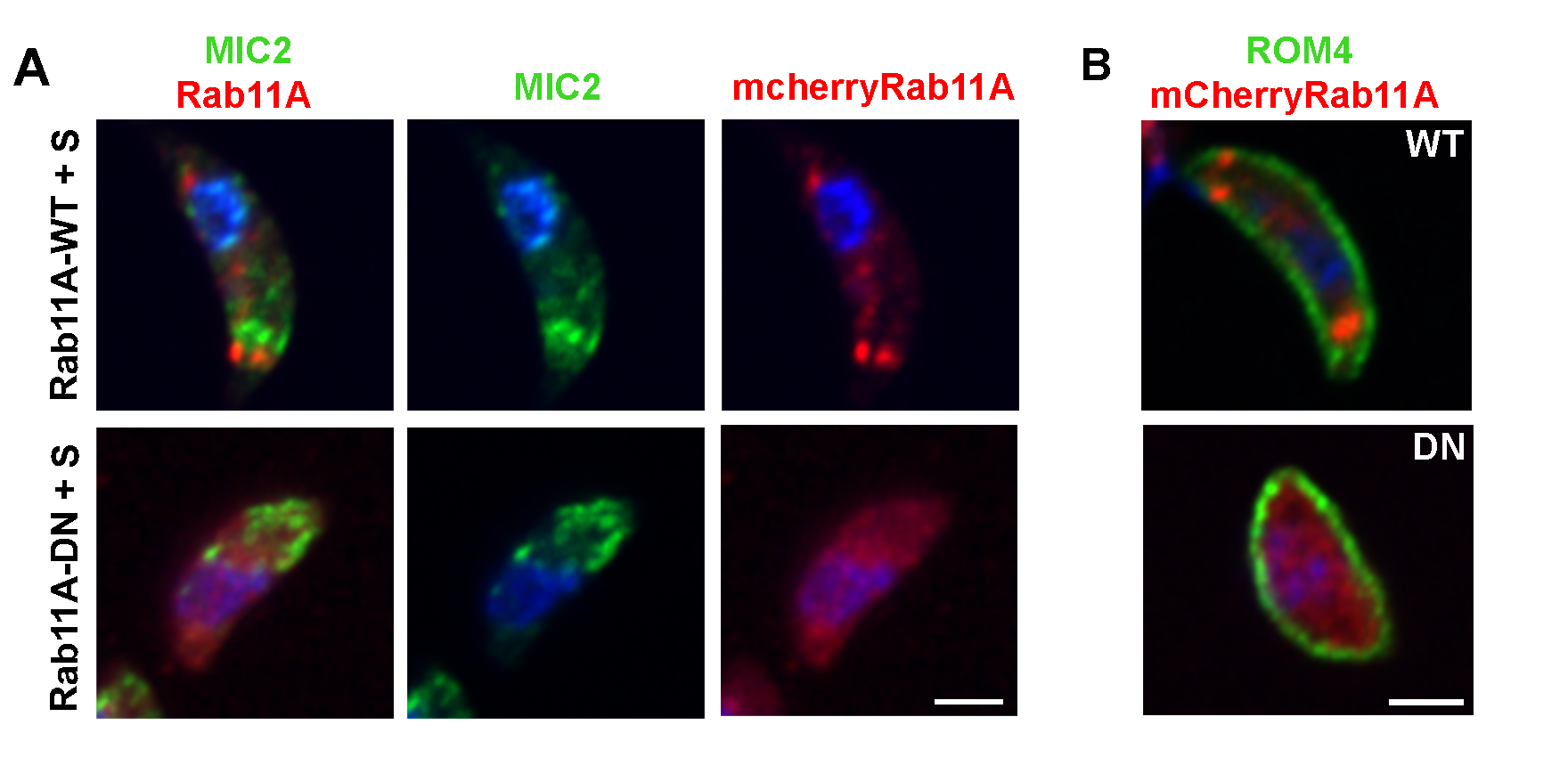

Supplement: S4 Fig — Immunofluorescence images showing a similar localization of apical MIC2-positive micronemes (A) and of the plasma membrane protein ROM4 (B) in Shield-1 induced Rab11A-WT and Rab11A-DN parasites. Bars: 2 μm. (TIF) [file ppat.1008106.s004.tif]

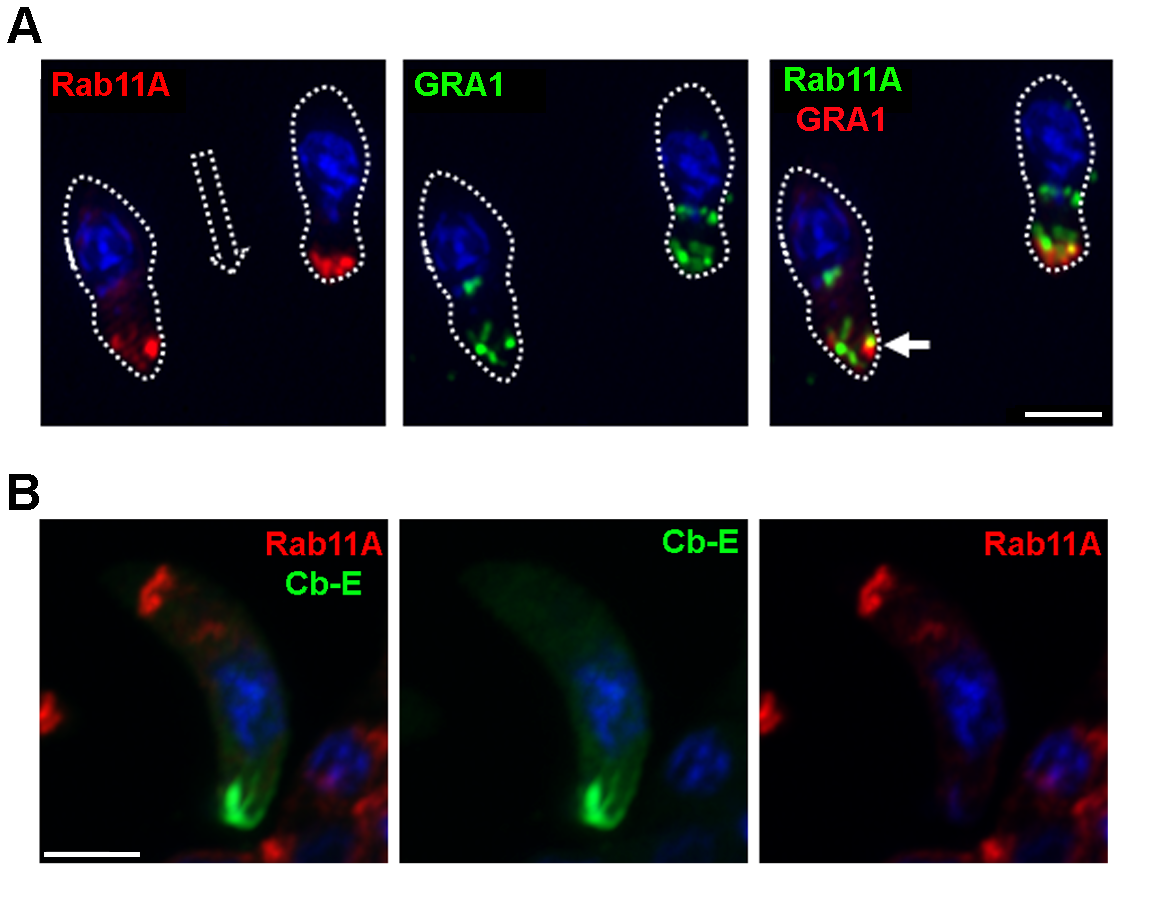

Supplement: S5 Fig — A- Immunofluorescence images showing the co-localization of the mcherryRab11A-positive signal (red) and GRA1-positive DG (green) at two apical foci in invading Rab11A-WT parasites. B- Immunofluorescence images showing the apical localization of mcherryRab11A (red) and the enrichment of actin (Cb-E, green) at the posterior pole of motile extracellular Rab11A-WT parasites. Bars: 2 μm. (TIF) [file ppat.1008106.s005.tif]
